# Physiological Markers of Arousal Change with Psychological Treatment for Insomnia: A Preliminary Investigation

**DOI:** 10.1371/journal.pone.0145317

**Published:** 2015-12-18

**Authors:** Christopher B. Miller, Simon D. Kyle, Christopher J. Gordon, Colin A. Espie, Ronald R. Grunstein, Anna E. Mullins, Svetlana Postnova, Delwyn J. Bartlett

**Affiliations:** 1 Centre for Integrated Research and Understanding of Sleep (CIRUS), NeuroSleep and Woolcock Institute of Medical Research, University of Sydney, Sydney, Australia; 2 Sleep & Circadian Neuroscience Institute, Nuffield Department of Clinical Neurosciences, University of Oxford, Oxford, United Kingdom; 3 Sydney Nursing School, University of Sydney, Sydney, Australia; 4 Department of Respiratory and Sleep Medicine, RPAH, Sydney Local Health District, Sydney, Australia; 5 School of Physics, University of Sydney, Sydney, Australia; University of Regensburg, GERMANY

## Abstract

**Objectives:**

The aim of this preliminary study was to evaluate if Sleep Restriction Therapy for insomnia is associated with modifications to physiological arousal, indexed through overnight measures of plasma cortisol concentrations and core body temperature.

**Methods:**

In a pre-to-post open label study design, eleven patients with chronic and severe Psychophysiological Insomnia underwent 5 weeks of Sleep Restriction Therapy.

**Results:**

Eight (73%) patients out of 11 consented completed therapy and showed a decrease in insomnia severity pre-to-post treatment (mean (SD): 18.1 (2.8) versus 8.4 (4.8); *p* = .001). Six patients were analyzed with pre-to-post overnight measures of temperature and cortisol. Contrary to our hypothesis, significantly higher levels of plasma cortisol concentrations were found during the early morning at post-treatment compared to baseline (*p* < .01), while no change was observed in the pre-sleep phase or early part of the night. Core body temperature during sleep was however reduced significantly (overall mean [95% CI]: 36.54 (°C) [36.3, 36.8] versus 36.45 [36.2, 36.7]; *p* < .05).

**Conclusions:**

Sleep Restriction Therapy therefore was associated with increased early morning cortisol concentrations and decreased core body temperature, supporting the premise of physiological changes in functioning after effective therapy. Future work should evaluate change in physiological variables associated with clinical treatment response.

**Trial Registration:**

Australian New Zealand Clinical Trials Registry ANZCTR 12612000049875

## Introduction

Models of chronic insomnia suggest hyperarousal and abnormal physiological functioning may be involved in the etiology of insomnia [1]. Supporting studies have profiled these factors through physiological assays including plasma cortisol concentrations and core body temperature measurements [2, 3]. Increased cortisol secretion has been found in insomnia compared to controls and may serve as a marker of increased hypothalamic–pituitary–adrenal (HPA) axis activation, particularly during the early part of the night [4]. Core body temperature, measured over the course of the night has also been found to be increased in insomnia [2, 3, 5]. Effective treatment for insomnia, therefore, may modify these markers of physiological arousal. Surprisingly, there has been a lack of studies documenting physiological measures after cognitive behavioural therapy (CBT) including plasma cortisol and core body temperature pre-to-post for insomnia [3, 6].

The hyperarousal theory is supported by evidence of altered thermoregulatory responses in patients with insomnia [7–9], particularly prior to sleep onset [10]. This augmentation of core body temperature results from an increased metabolic rate which is consistently elevated across the 24-hour period compared with controls [11]. The decline in core temperature prior to sleep onset may be impaired in insomnia patients, possibly reflecting impaired heat dissipation at sleep onset. Supporting an impaired heat dissipation hypothesis, insomnia patients had attenuated vasodilation of their finger compared with healthy sleeping controls in response to mild thermal challenges [12]. Changes in core body temperature and metabolic rate is also influenced by sympathetic activity and elevated in those with insomnia compared to controls [2, 3, 5]. As a result, core body temperature appears to be a maker of sympathetic activity and overall physiological hyperarousal in insomnia. Therefore, reductions in core body temperature in insomnia may reflect improvements in insomnia severity [3].

Rodenbeck and colleagues [13] reported a significant reduction in night time plasma cortisol secretion after three weeks of Doxepin compared to placebo in a crossover study with 10 insomnia patients. Another, study (*n* = 5) reported reduced salivary cortisol levels during Sleep Restriction Therapy (SRT: a standalone behavioural component of overall CBT for insomnia), suggesting that behavioural treatment, may also alter cortisol concentrations [14]. Both studies attributed changes to reduced HPA-axis activation. Consequently, it remains unclear if reduction in HPA-axis activation is a mechanism of therapeutic action. Treatment outcomes including plasma cortisol remain an untested outcome of CBT [15]. SRT may improve arousal processes by reducing sleep onset times and time awake during the night, resulting in improved sleep efficiency (%SE: proportion of time asleep to the amount of time spent in bed) and overall insomnia severity [16].

In this preliminary study, we evaluate whether or not SRT is associated with modification to physiological arousal. We hypothesised that SRT will reduce physiological arousal (i.e. reduce early night plasma cortisol, core body temperature).

## Materials and Methods

### Patients

**Fig 1:**
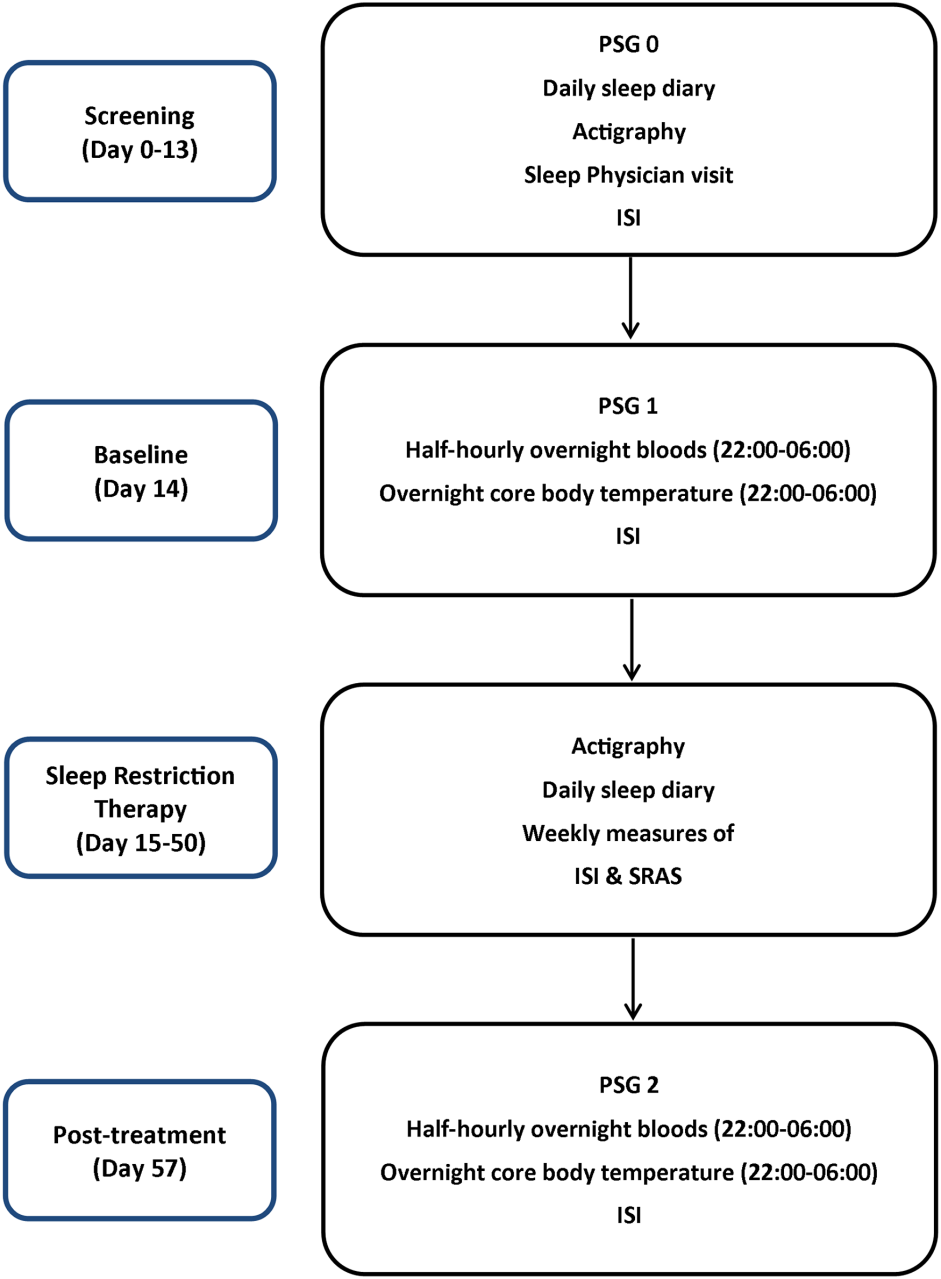
Displays the study protocol. Prior to study enrolment, patients were examined for sleep disorders through 1 night of polysomnography (PSG) evaluation, sleep diary and actigraphic data. Prior to therapy, a baseline assessment of sleep with half hourly blood and minutely core body temperature measures was implemented. Sleep restriction therapy was initiated over the course of 5 weeks with daily sleep diaries, actigraphic recordings and weekly questionnaire assessments including: the Insomnia Severity Index: ISI; Sleep Restriction Adherence Scale: SRAS. After 6 weeks, patients returned for a post-treatment overnight study again with overnight measures of both blood and temperature.

Patients naïve to CBT were recruited from a larger insomnia phenotyping study (*n* = 100: from patient numbers 24–66) [Clinical Trial Registration (Australian New Zealand Clinical Trials Registry: ANZCTR):12612000049875]. Patients who responded to advertisements, self-referred into a tertiary level sleep clinic from the general population into this within-subjects pre-to-post open label trial (patients acted as their own controls) from October, 2012 until November, 2013. Psychophysiological Insomnia (PI), the most common insomnia sub-type characterised by learned anxiety and heightened arousal of sleep, was determined by a Sleep Physician for sleep initiation and/or maintenance subtypes only [17] as early morning awakening insomnia may not respond as well to SRT [18]. Inclusion criteria was the primary complaint of insomnia, a subjective sleep disturbance of 3 nights per week for at least 3 months, sleep onset latency (SOL) and/or wake-time after sleep-onset (WASO) >30 minutes, and a specific daytime impairment attributed to disturbed sleep [17]. For screening, patients completed sleep diaries and wore actigraphs for at least 1 week for a baseline assessment of sleep. A full polysomnographic (PSG) sleep assessment was also undertaken. Exclusionary criteria included current or unstable medical, co-morbid sleep or psychiatric conditions, mean Apnea Hypopnea Index (AHI) scores of >10, Periodic Limb Movements (PLMS) arousal index scores of >5 or minimum oxygen saturations of <90% during the night and reported chronic hypnotic use (>3 times per week). Two patients with occasional hypnotic use (1–3 times per week) were included but were asked to refrain from any hypnotic medication (under guidance from the Sleep Physician) throughout the study. Insomnia Severity Index (ISI) scores were used to define insomnia severity and subtype in conjunction with clinical evaluation, only those with an ISI score ≥15 (the cut off for clinical insomnia [19])—see Fig 1. for an overview of the study procedures. All data were collected at the Woolcock Institute of Medical Research, University of Sydney, Australia. This was a preliminary range finding study as a specific sample size could not be statistically calculated.

### Sleep Restriction Therapy

SRT comprised one 40-minute individual face-to-face session using Spielman et al., (1987) initial instructions [20]. In line with previous guidance, a minimum time in bed (TIB: sleep window) was set at 5 hours [16, 21]. The sleep window was anchored with a morning wake-up time to suit individual patient daytime commitments. The therapist then calculated the initial go to bed ‘threshold time’. Patients were instructed to not go to bed until after the threshold time and to only go to bed if they felt ‘sleepy tired’. The intervention was standardised and supported by a brief set of digital slides (8 in total) and an information manual (11 pages, in plain English). Up to 5 weekly telephone calls (each 5–10 min) were provided to review (%SE) and titrate the sleep window. For titration, TIB was modified on a weekly basis. Changes were made to TIB incrementally, depending on the achievement of ‘good sleep efficiency’: 85–89% no change, >90% increase by 15 min, <85% decrease by 15 min [18]. Patient negotiation was taken into account regarding achievable sleep scheduling, but only if the patient was experiencing particular difficulty in adhering to the schedule. Time out of bed was rarely changed. In nearly all cases time to bed was modified in line with previous instructions [18]. Patients were also asked to avoid napping as much as possible during the study unless they required a nap (20 minutes maximum) as a counter measure to extreme daytime sleepiness. No other CBT components were addressed. The first author (CBM) acted as the therapist in this study. This study was conducted according to the principles expressed in the Declaration of Helsinki and reviewed and approved by the Royal Prince Alfred Hospital Ethics Review Committee, Sydney, Australia (Protocol No X11-0408 & HREC/11/RPAH/634); clinical trial registration number: Australian New Zealand Clinical Trials Registry (ANZCTR) 12612000057886. All patients gave written informed consent.

### Adherence

The sleep restriction adherence scale (SRAS) was completed on a weekly basis throughout therapy (first 5 weeks) and served as a primary measure of adherence. The scale is based on the Medical Outcomes Study general Adherence scale (MOS-A: [22]), and modified to probe adherence to different aspects of SRT. Total SRAS scores range from 5–30, with higher scores indicative of greater levels of adherence [23]. Preliminary psychometric evaluation of the SRAS, with 42 insomnia patients undergoing SRT, revealed high internal consistency (Cronbach’s α = 0.92; range of item-deletion α = 0.89–0.93, mean α = 0.91). Sleep diary data (TIB) was also checked for adherence to the sleep window.

### Polysomnography

PSG was used as a screening tool for other sleep disorders on day 0 (see Fig 1), as well as before and after SRT (at 6 weeks) for objective sleep architecture and continuity variables. A time frame of 6 weeks from the start of therapy was selected as previous research showed sleep duration was markedly reduced relative to baseline for the first 3-weeks of SRT [16]. For all 3 PSG assessments, a research montage was used involving 10-lead electroencephalographic [EEG: Fpz (neutral), F3, Fz, F4, C3, Cz, C4, Cz-Pz (Reference), Pz, O1, Oz, O2], electrooculographic (EOG: horizontal and vertical), electrocardiographic (ECG) and electromyographic (EMG: submental) recordings. Data were recorded on an Embla Titanium ambulatory recorder (Mortara, Milwaukee, USA) and scored simultaneously by 2 experienced sleep scorers according to AASM criteria [24].

### Overnight Cortisol and Core body temperature data collection

Overnight blood and core body temperature collection was performed at baseline and after therapy during the PSG nights. To standardise data collection, all patients were set-up and in bed from 21:00 on both nights of data collection. Patients however remained awake until they felt sleepy tired, ready to initiate sleep and then decided on their own lights out time. Blood was collected every half-hour from 22:00 until the last sample at 06:00 (17 samples in total). An intravenous catheter was inserted under sterile conditions into the cubital fossa vein and attached to 140cm intravenous tubing to sampling port in another room, so as to minimise sleep disruption [4]. If not already awake patients were awoken after the final sample at 06:00. Samples were placed on ice for 5 minutes, centrifuged at 5,000 RPM for 10 minutes at 3°C and stored at -80°C until analysis by enzyme immunoassay was undertaken (Cayman Chemical Company, Ann Arbor, Michigan; limit of detection: 80% B/B0: 35 pg/ml, Sensitivity: 50% B/B0: 180 pg/ml). Cortisol was analysed on site at the Woolcock Institute of Medical Research (Sydney, Australia) as described previously [4, 25]. Core body temperature was sampled once a minute from 22:00 onwards until 06:00 using a previously validated [26] ingestible core body temperature pill (Phillips Respironics, Bend, Oregon, USA). To minimise the variability in thermoregulatory responses associated with the menstrual cycle, all females that were pre-menopausal were tested during the first 10 days of the follicular phase of the menstrual cycle [27]. Women who were post-menopausal and asymptomatic (*n* = 6) did not have restricted testing days. Initially, this study was designed to include a 24-hour constant routine schedule pre-to-post SRT for each patient however; these were reduced to two overnight assessments only due to cost.

### Self-report sleep diary & insomnia severity

Patients completed daily sleep diaries for at least 1 week prior to therapy and for 6 weeks from treatment start for the following outcomes: SOL; WASO; Number of Awakenings (NOAW); Total Sleep Time (TST); TIB; %SE; Sleep Quality (SQ). The ISI [19] was completed before, during and after therapy to monitor global differences with treatment.

### Statistical analysis

For all patient outcome variables, boxplots and histograms were used to check data for normality and outliers. Variables were graphed and visually checked for individual patient changes pre-to-post therapy. For cortisol concentrations and core body temperature, data were analysed by linear mixed model using Statistical Package for the Social Sciences (SPSS) software (IBM v 22.0.0; IBM, Amonk, NY, USA) to take account of fixed effects including time (pre-to-post therapy) and sample collection over the course of the night (22:00–06:00). Random effects were included to account for between-subject variation and were used for the intercepts. Pairwise comparisons with Bonferroni adjustment were based on estimated marginal means and used to investigate primary outcomes. Paired *t*-tests were employed to profile differences pre-to-post therapy on all other variables. Effect size scores were calculated where appropriate [28, 29].

## Results and Discussion

**Fig 2:**
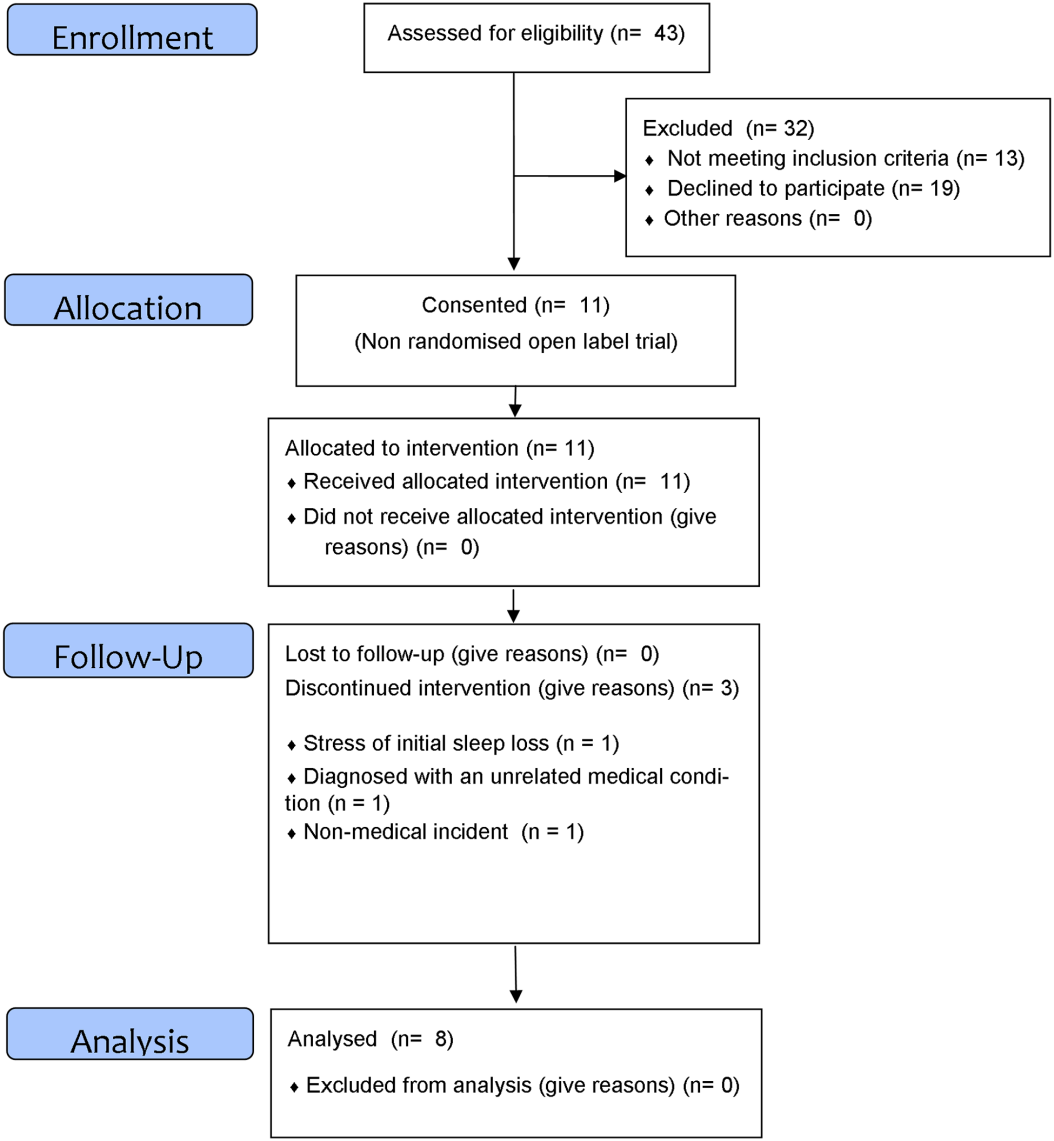
Study CONSORT flow diagram.

Eight out of eleven patients (2 male: mean age 45.5 years, range 25–60) completed this study (see Fig 2). Full pre-to-post blood and core body temperature data are reported for 6 out of the 8 completed patients. Baseline blood (due to problems inserting the intravenous catheter) and temperature collection (due to battery malfunction) failed in another subject. At post-treatment, there was a similar problem with catheter insertion in 1 patient. One patient was unable to ingest the core body temperature pill due to previous abdominal surgery. Average insomnia duration was 11.3 years (range from 1–40). For therapy adherence, mean SRAS scores were high (>20) and sleep diaries also suggested therapy was adhered to. Mean objective actigraphic recordings of TST decreased significantly between Baseline and Week 1 (*p* < .05) and remained lower for the 5 weeks of SRT, mirroring previous objective sleep findings [16] and suggesting adherence [Baseline mean (SE) = 422.2 (19.5); Week 1 = 335.9 (19.4); Week 2 = 342.4 (19.5); Week 3 = 364.2 (19.5); Week 4 = 355.1 (19.4); Week 5 = 369.4 (19.4)].

### Treatment Response

Mean ISI scores decreased significantly pre-to-post therapy (mean (SD): 18.1 (2.8) versus 8.4 (4.8); *p* = .001). Primary sleep diary variables were analysed to test differences pre-to-post SRT (see Table 1). SOL displayed a trend, suggesting a reduction (*d* = 0.6). Both WASO and the NOAW’s decreased significantly (*p* < .05) with a large effect (*d* = 1.6, 0.9 respectively). Sleep efficiency (%SE) and ratings of sleep quality both increased significantly (*p* < .01) with a large effect (*d*≥1.5) whereas TST did not significantly change (mean (SD) = 335.9 (53.2) versus 353.4 (72.8); *p* = .27, *d* = 0.3). There were no significant changes for objectively defined (PSG) sleep parameters although TST increased by approximately 40 minutes, but this was not significant (313.7 (54.2) versus 353.6 (40.9); *p* = .10, *d* = 0.8). There were no serious adverse events reported for this trial.

**Table 1:** Sleep diary variables.

Mean scores (+/-SD)	Baseline	Week 6	Baseline versus week 6	*p*-value	Effect size (*d*)
SOL	51.6 (58.9)	23.0 (22.0)	-28.7 (14.7)	.10	0.6
WASO	73.9 (54.7)	11.2 (9.8)	**-62.7 (59.1)** *	.03	1.6
NOAW	2.1 (1.4)	1.0 (0.9)	**-1.1 (0.28)** *	.01	0.9
TST	335.9 (53.2)	353.4 (72.8)	17.5 (38.1)	.27	0.3
TIB	526.0 (36.1)	427.5 (57.2)	**-98.6 (66.5)** *	< .05	2.1
%SE	64.7 (12.3)	82.7 (12.5)	**10.6 (4.0)** *	< .05	1.5
SQ	0.6 (0.3)	1.24 (0.4)	**0.66 (0.1)** **	< .01	1.8

Self-report sleep diary variables pre-to-post Sleep Restriction Therapy for Insomnia. The left hand side of the table displays mean sleep diary data at baseline and 6 weeks after starting Sleep Restriction Therapy (*n* = 8). The right hand side of the table examines within subject differences between baseline and week 6. SOL: Sleep Onset Latency; WASO: Wake-time After Sleep Onset; NOAW: Number of Awakenings; TST: Total Sleep Time; TIB: Time in Bed; %SE: Sleep Efficiency; SQ: Sleep Quality.

* = *p* < .05;

** = *p* < .01.

### Circadian oscillation of outcome variables

**Fig 3:**
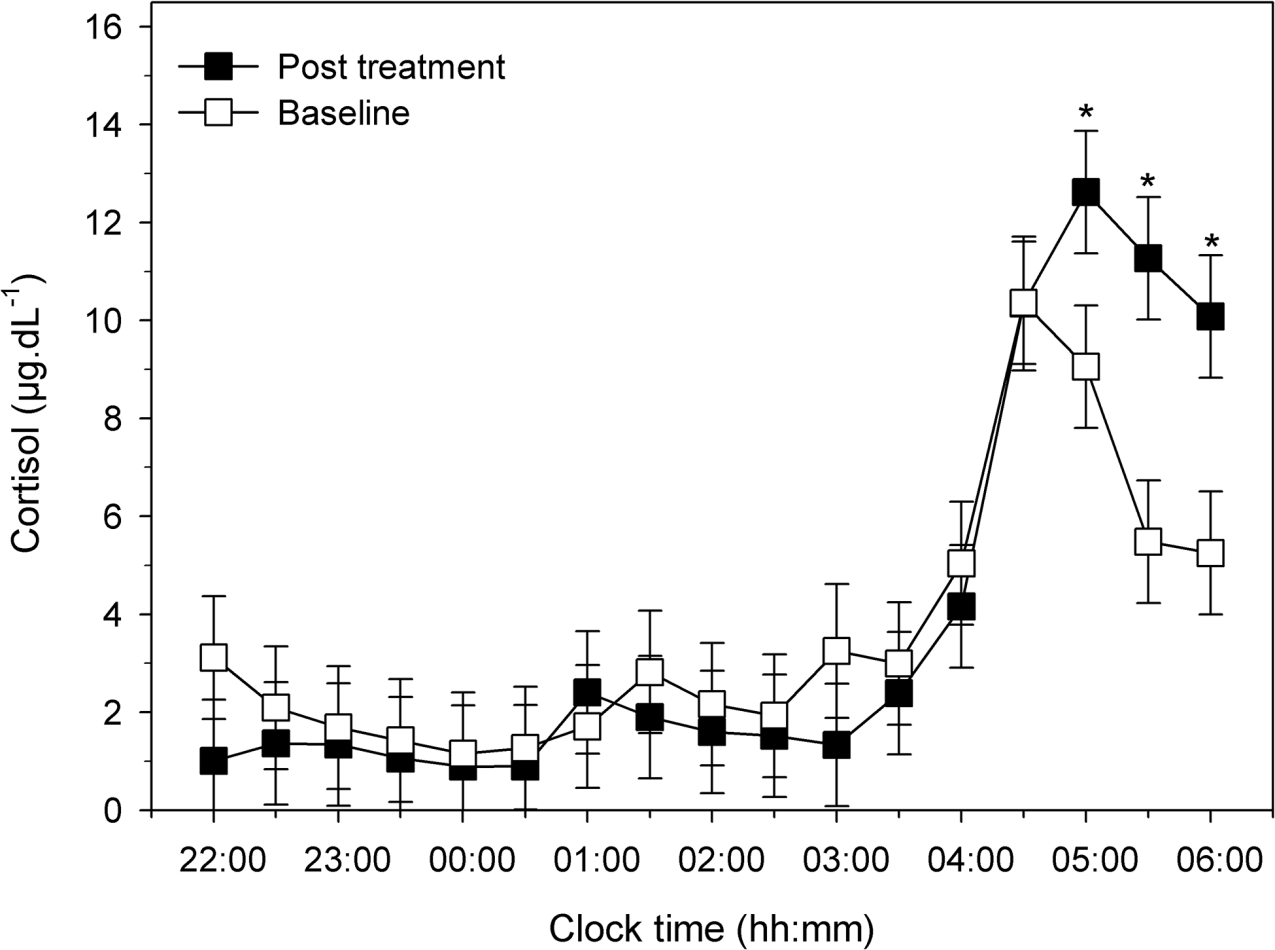
Cortisol concentrations across the night pre-to-post sleep restriction therapy. Mean nocturnal cortisol secretion (*n* = 6) for each sample collection time point (baseline and post treatment) is displayed over the course the night (22:00–06:00). Error bars indicate one standard error of the mean. Cortisol (μg/dL^-1^). (*) = *p* < .05.

**Fig 4:**
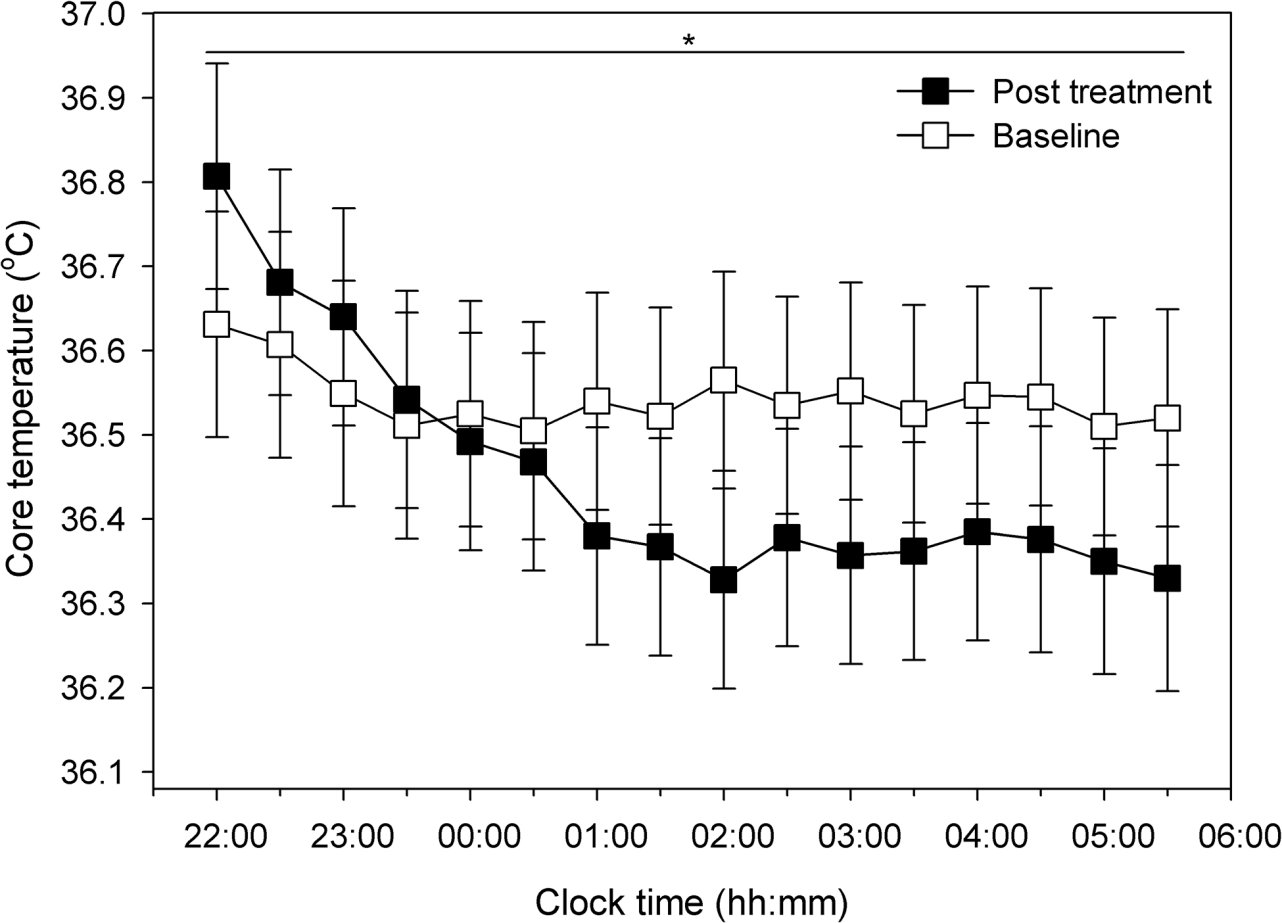
Core body temperature across the night pre-to-post sleep restriction therapy. Mean nocturnal core body temperature in degrees Celsius (°C) (*n* = 6) for each sample collection time points (baseline and post treatment) is displayed over the course the night (22:00–06:00). Error bars indicate one standard error of the mean. (*) = *p* < .05.

Prior to the primary analysis, mean plasma cortisol concentrations and core body temperature data (collapsed into 30 minute bins) were first checked for nocturnal circadian oscillation across the night (22:00–06:00, *n* = 6). As expected, a significant effect for time was found for both cortisol secretion (μg/dL^-1^) (F (16, 163.06) = 14.86; *p* < .01) and core body temperature (°C) (F (15, 145.02) = 1.94; *p* = .02) (see Figs 3 and 4).

### Physiological Arousal

Regarding our hypothesis, to determine if SRT reduces overall physiological arousal (i.e. reduces cortisol and core body temperature), samples were evaluated for mean pre-to-post differences in patients (*n* = 6). For cortisol, no significant overall differences were found pre-to-post therapy (mean and 95% confidence intervals [CI]: 3.58 [2.6, 4.5] versus 3.89 [2.9, 4.9], F (1, 163.03) = .57; *p* = .45). Next, we tested for differences pre-to-post SRT on mean core body temperature which displayed a significant decrease after therapy (36.54 [36.28, 36.81] versus 36.45 [36.19, 36.72], F (1, 145.02) = 9.69; *p* < .01).

To evaluate within-subject differences across the sleep period pre-to-post therapy, a sample (for each half hour sample during the night: 22:00–06:00) x time (pre-to-post) phase interaction was used (F (16, 163.06) = 1.68; *p* = .055). Pairwise comparisons revealed a significant increase at the following sample time points pre-to-post therapy: 05:00 (mean [CI] = 9.1 [6.6, 11.5] versus 12.6 [10.2, 15.1]; *p* = .04); 05:30 (5.5 [3.0, 8.0] versus 11.3 [8.8, 13.7]; *p* < .01); 06:00 (5.3 [2.8, 7.7] versus 10.1, [7.6, 12.6]; *p* = .01) (see Fig 3). We also examined cortisol and core body temperature relative to mean sleep onset time. With the same analysis we found similar results (see S1 and S2 Figs).

### Circadian timing of core the body temperature

Lastly, an analysis of individual changes in temperature rhythm pre-to-post study (with best fitting sinusoidal curves to temperature data curves (according to the following formula: *a*+b*sin(c*x+*d*), where *a* is the offset, *b* is the amplitude, *c* is close to *pi*/12 to achieve 24hr periodicity and *d* is the phase [30] did not reveal any circadian phase or periodicity changes.

In this preliminary study, Insomnia severity (ISI) and with sleep diary outcomes reduced significantly pre-to-post SRT. These findings support the efficacy of SRT, as previously found in controlled and uncontrolled trials [31, 32]. Therapy was adhered to as indicated by patient self-report measures (SRAS). This is the first study to our knowledge to profile nocturnal measures of physiological arousal before and after a behavioural intervention for insomnia. We hypothesised a reduction in early night time plasma cortisol concentrations and core body temperature in line with a reduction in physiological arousal [3]. While we found evidence that SRT is associated with a reduction in core body temperature, contrary to our first hypothesis, early night time plasma cortisol concentrations did not change. Secondly, we found no evidence of an overall phase change in the nadir of core body temperature.

Physiological arousal is thought to be both a precipitating and perpetuating factor in the development and maintenance of chronic insomnia [33]. Previously, core body temperature was increased in insomnia compared to controls by approximately 0.2–0.3°C under controlled conditions [3, 5]. In this study, core body temperature decreased significantly, as expected when physiological hyperarousal (excluding cognitive arousal) is reduced; especially in the pre-sleep period, as shown in Fig 4 [12, 34, 35]. This modest mean reduction of 0.09°C suggests SRT reduces physiological arousal in insomnia after 6 weeks. The clinical significance of this temperature reduction however remains to be determined; a longer follow-up assessment point (6 months) may be required to detect sustained decreases that are comparable to between group differences.

It could be argued that sleep loss from SRT could be responsible for this finding. Actigraphy however indicated that patients were obtaining approximately six hours of sleep on average during weeks 3–5 of therapy. Mild sleep deprivation and five 24-hour constant routine schedules did not alter core temperature in healthy controls [36, 37].

It was expected that nocturnal cortisol concentrations would reduce with effective treatment as previous research has indicated increased cortisol secretion in insomnia compared to controls [3, 4]. SRT did not reduce cortisol concentrations. Early morning cortisol concentrations were higher post therapy, suggesting normalisation of the cortisol awakening response previously reported in untreated patients with insomnia compared to controls [38]. Increased morning cortisol concentrations may be clinically important as adverse health effects and psychiatric disorders (including depression) have been associated with a blunted awakening response [39]. Increased morning alertness in our previous, SRT study suggests that it may drive optimal daytime functioning through increased morning cortisol and reductions in overall insomnia severity [23].

The nadir of core body temperature did not advance after therapy. This may have been due to the lack of circadian amplitude at baseline which made it difficult to identify the nadir for curve fitting but was more easily defined after therapy (see Fig 4). A faster decline in core body temperature was visually apparent during the first two hours of sleep initiation at post treatment compared to baseline. This decline in temperature appears suggestive of change in circadian amplitude and not circadian phase after therapy. Without 24-hour data under constant routine conditions, conclusions are speculative. The rate of decline post-therapy of core body temperature also suggests that sleep initiation may have been enhanced to reflect the thermophysiological cascade required prior and following sleep onset [34].

The lack of a control group is a clear limitation of this preliminary work, as patients may have improved through a placebo/non-specific response. As there is no comparative reference point, it is not possible to state patients are abnormal for cortisol or core body temperature at baseline and may not reflect previous insomnia case-control studies [3–5]. A 6 month follow-up assessment would have assessed whether changes are maintained over time. Female menstrual patterns may impact core body temperature; however only 1 female was pre-menopausal and reported a regular 28-d menstrual cycle and post-menopausal women may have more stable temperature rhythms [40]. The one menstruating female was tested in the follicular phase at baseline prior to SRT and during follicular post therapy. Further trials with 24-hour constant routine conditions or dim light melatonin onset assessment pre-to-post therapy are now required to verify this finding by eliminating potentially confounding exogenous variables for masking effects. TIB was limited during the acute phase of the intervention (first 5 weeks) however patients continued to use sleep diaries to self-titrate the sleep window for one week prior to the follow-up overnight assessment. TIB was gradually increased by at least week three on average until week five as part of therapy. At the post treatment assessment six weeks after therapy, changes in cortisol and temperature were potentially reflecting increased %SE and reduced insomnia severity. Catch-up sleep may be responsible for findings at the post treatment assessment; however patients averaged 353 minutes of in-lab PSG-defined sleep, whilst actigraphy showed patients were obtaining similar amounts of sleep at home prior to the week 6 follow-up (approximately 6 hours on average during weeks 3–5). This goes against the notion of catch-up sleep being responsible for changes in cortisol and temperature. The limitations of a small sample size and the increased risk of a Type-II statistical error are apparent. Studies with larger numbers that relate potential changes in physiological variables (including temperature and cortisol concentrations) to clinical response are required. Lastly, the use of an overnight intravenous catheter may have disturbed sleep and arousal levels during the baseline assessment and patients may have adapted to this at the post-treatment assessment. Results are therefore necessarily preliminary until verified by more robust randomised controlled trial study design.

### Conclusion

This is the first preliminary study to specifically profile nocturnal physiological parameters relating to arousal after a behavioural intervention for the treatment of insomnia. SRT was associated with overall reduced core body temperature and increased plasma cortisol concentrations in the later third of the night. Future work should evaluate change in physiological outcomes after therapy for insomnia.

## Supporting Information

S1 Fig Cortisol concentrations across the night pre-to-post sleep restriction therapy corrected for sleep onset. Mean nocturnal plasma cortisol concentrations across the night pre-to-post sleep restriction therapy. Mean nocturnal cortisol secretion (*n* = 6) for each sample collection time point (baseline and post treatment) is displayed over the course the night for each hour relative to sleep onset. Dashed vertical line represents sleep onset time. Error bars indicate one standard error of the mean. Cortisol (μg/dL^-1^). (*) = *p* < .05.
(TIF)

S2 Fig Core body temperature across the night pre-to-post sleep restriction therapy corrected for sleep onset. Mean core body temperature across the night pre-to-post sleep restriction therapy. Mean core body temperature in degrees Celsius (°C) (*n* = 6) for both sample collection time points (baseline and post treatment) is displayed over the course the night for each hour relative to sleep onset. Dashed vertical line represents sleep onset time. Error bars indicate one standard error of the mean. (*) = *p* < .05.
(TIF)

S1 File Study TREND statement checklist document. (DOCX)

S2 File Study protocol document. (DOC)

S1 Data Includes cortisol data to produce Fig 3, core body temperature data to produce Fig 4, cortisol data to produce S1 Fig and core body temperature to produce S2 Fig. (ZIP)
