# Supplementary figures and images for: Physiological Markers of Arousal Change with Psychological Treatment for Insomnia: A Preliminary Investigation
Source: PLoS One. 2015 Dec 18;10(12):e0145317. doi: 10.1371/journal.pone.0145317 (PMC4689577; doi:10.1371/journal.pone.0145317)

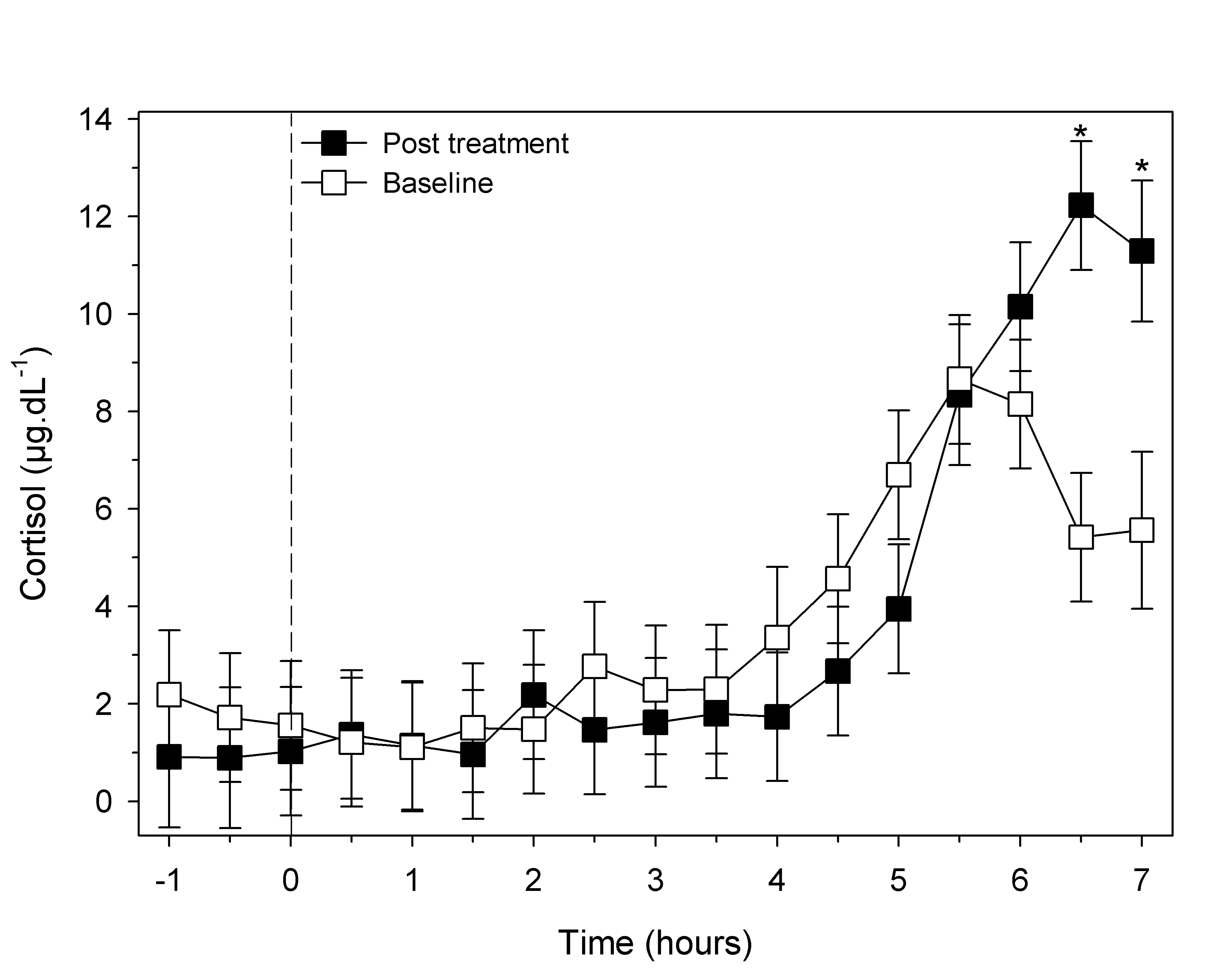

Supplement: S1 Fig — Mean nocturnal plasma cortisol concentrations across the night pre-to-post sleep restriction therapy. Mean nocturnal cortisol secretion (n = 6) for each sample collection time point (baseline and post treatment) is displayed over the course the night for each hour relative to sleep onset. Dashed vertical line represents sleep onset time. Error bars indicate one standard error of the mean. Cortisol (μg/dL-1). (*) = p < .05. (TIF) [file pone.0145317.s001.tif]

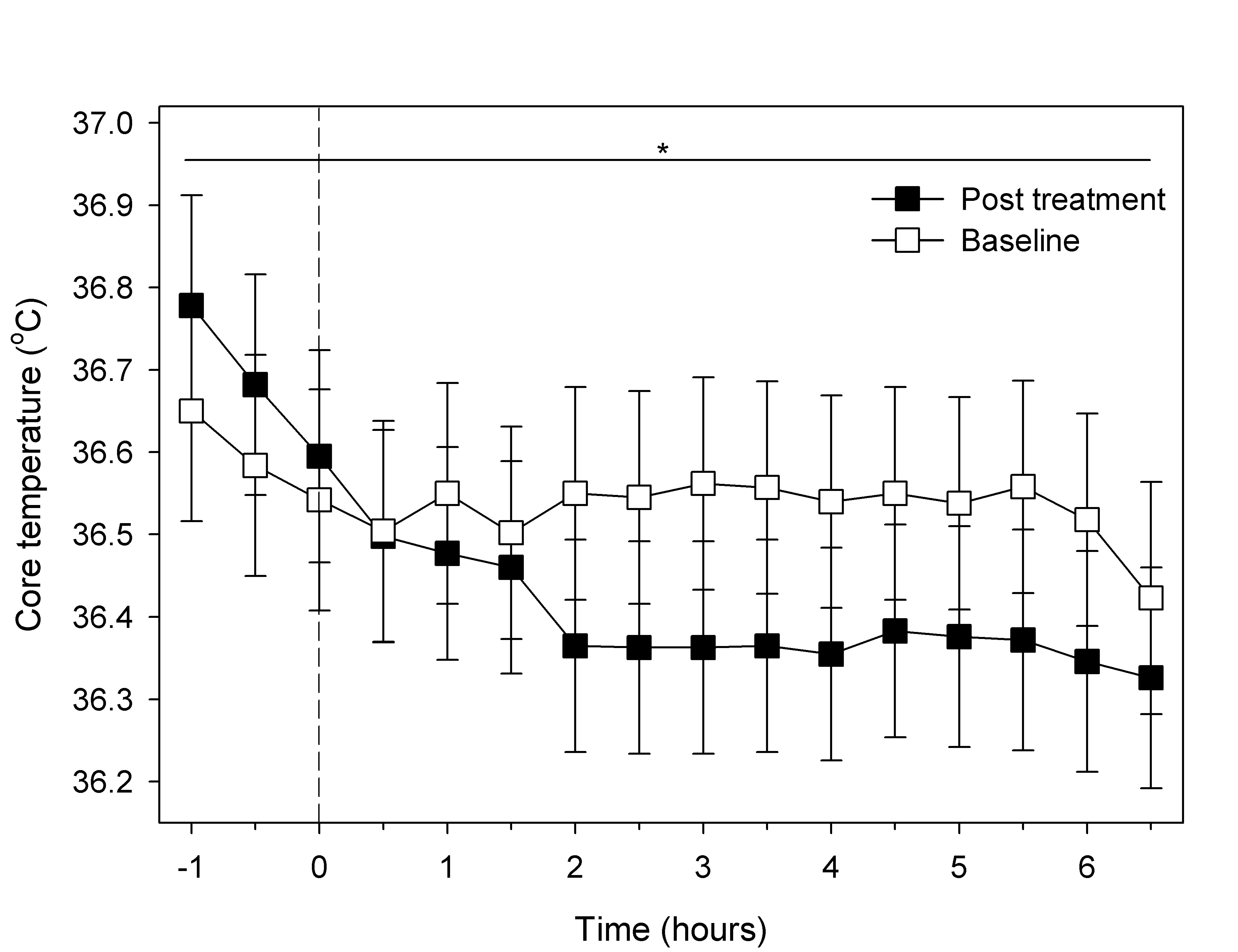

Supplement: S2 Fig — Mean core body temperature across the night pre-to-post sleep restriction therapy. Mean core body temperature in degrees Celsius (°C) (n = 6) for both sample collection time points (baseline and post treatment) is displayed over the course the night for each hour relative to sleep onset. Dashed vertical line represents sleep onset time. Error bars indicate one standard error of the mean. (*) = p < .05. (TIF) [file pone.0145317.s002.tif]
