# Supplementary material for: Physiological Markers of Arousal Change with Psychological Treatment for Insomnia: A Preliminary Investigation
Source: PLoS One. 2015 Dec 18;10(12):e0145317. doi: 10.1371/journal.pone.0145317 (PMC4689577; doi:10.1371/journal.pone.0145317)
Supplement: S2 File — (DOC) [file pone.0145317.s004.doc]

# SRT Testing Protocol

| **Time** | **Activity** | **Staff Member** | **Notes** |
| --- | --- | --- | --- |
| One week Before | Consent, Assign Sleep Dairies & actiwatch | Chris |  |
| *First Sleep Study* | | | |
| 18:00 | Arrive at the Woolcock | Technician |  |
| 18:15 | Take blood pressure & blood | Research Nurse |  |
| 19:15 | Prepare for Sleep Study | Technician |  |
| 22:00 | Go to bed | Technician |  |
| *Morning* | | | |
| 6:00 | Wake up | Technician |  |
| 7:00 | Leave Woolcock and return that evening | Technician |  |
| *First Constant Routine* | | | |
| 17:00 | Arrive at the Woolcock | Technician |  |
| 17:15 | Take blood pressure & set-up blood sampling | Research Nurse |  |
| 18:00 -  18:00 | 24 Hourly Assessments for Blood, Computer tasks, & Questionnaires | Technician |  |
| 18:00 | End of 24 hour routine | Research Nurse |  |
| 19:00 | Leave Woolcock in taxi | Technician |  |
| *Ten day gap* | | | |
| 18:00 – 19:00 | Arrive at the Woolcock for Sleep Therapy 1, Computer tasks, & Questionnaires | Delwyn & Chris |  |
| *Seven day gap* | | | |
| 18:00 – 19:00 | Arrive at the Woolcock for Sleep Therapy 2, Computer tasks, & Questionnaires | Delwyn & Chris |  |
| *Seven day gap* | | | |
| 18:00 – 19:00 | 15 minute treatment telephone call 1 | Chris |  |
| *Seven day gap* | | | |
| 18:00 – 19:00 | Arrive at the Woolcock for Sleep Therapy 3, Computer tasks, & Questionnaires | Delwyn & Chris |  |
| *Seven day gap* | | | |
| 18:00 – 19:00 | 15 minute treatment telephone call 2 | Chris |  |
| *Seven day gap* | | | |
| 18:00 – 19:00 | Arrive at the Woolcock for Sleep Therapy 4, Computer tasks, & Questionnaires | Delwyn & Chris |  |
| *Seven day gap* | | | |
| *Second Sleep Study* |  |  |  |
| 18:00 | Arrive at the Woolcock | Technician |  |
| 18:15 | Take blood pressure & blood | Research Nurse |  |
| 19:15 | Prepare for Sleep Study | Technician |  |
| 22:00 | Go to bed | Technician |  |
| *Morning* |  |  |  |
| 6:00 | Wake up | Technician |  |
| 7:00 | Leave Woolcock and return that evening | Technician |  |
| *Second Constant Routine* |  |  |  |
| 17:00 | Arrive at the Woolcock | Technician |  |
| 17:15 | Take blood pressure & set-up blood sampling | Research Nurse |  |
| 18:00 -  18:00 | 24 Hourly Assessments for Blood, Computer tasks, & Questionnaires | Technician |  |
| 18:00 | End of 24 hour routine | Research Nurse |  |
| 19:00 | Leave Woolcock in taxi | Technician |  |
| End of Main study | Retrieve actiwatch | Chris |  |
| *Nine week gap* |  |  |  |
| Follow-up assessment | Arrive at the Woolcock Computer tasks, & Questionnaires | Chris |  |
| End |  |  |  |
